# Protective effects of *Pelargonium graveolens* (geranium) oil against cefotaxime-induced hepato-renal toxicity in rats

**DOI:** 10.3389/ftox.2024.1489310

**Published:** 2024-12-04

**Authors:** Shaimaa M. Azzam, Heba M. A. Elsanhory, Ahmed H. Abd El-Slam, Marwa S. M. Diab, Halima Mohamed Ibrahim, Abdalrahman Mohammed Yousef, Fatma Mahmoud Sabry, Ebtihal Y. Khojah, Somaiah A. Bokhari, Gad Elsayed Mohamed Salem, Marwa Saad Zaghloul

**Affiliations:** ^1^ Department of Biochemistry, Egyptian Drug Authority (EDA), Formerly National Organization for Drug Control and Research (NODCAR), Giza, Egypt; ^2^ Pharmacology and Toxicology Department, Faculty of Pharmacy, Sinai University, El Ismailia, Egypt; ^3^ Department of Forensic Medicine and Clinical Toxicology, Faculty of Medicine, Al-Azhar University, Cairo, Egypt; ^4^ Cell Biology and Histology, Molecular Drug Evaluation Department, Egyptian Drug Authority (EDA) Formerly National Organization for Drug Control and Research (NODCAR), Giza, Egypt; ^5^ Department of Physiology, Egyptian Drug Authority (EDA), Formerly National Organization for Drug Control and Research (NODCAR), Giza, Egypt; ^6^ Pharmacology Department, Egyptian Drug Authority (EDA), Formerly National Organization for Drug Control and Research (NODCAR), Giza, Egypt; ^7^ Department of Food Sciences and Nutrition, College of Science, Taif University, Taif, Saudi Arabia; ^8^ Pharmaceutical Care Department, Maternity and Children Hospital, Mecca, Saudi Arabia; ^9^ Department of Microbiology, Egyptian Drug Authority(EDA), Formerly National Organization for Drug Control and Research (NODCAR), Giza, Egypt

**Keywords:** geranium oil, cefotaxime, hepatotoxicity, oxidative stress, antioxidant defense, MAPK, Nrf2, nephrotoxicity

## Abstract

Cefotaxime is a broad-spectrum antibiotic targeting Gram-negative bacteria used for diverse infections, but it can be toxic to the stomach, liver, and kidneys. This study explored the protective effects of geranium oil against cefotaxime-induced hepatotoxicity and nephrotoxicity in rats, employing biochemical, histopathological, and immunohistochemical evaluations. Thirty rats were divided into five groups of six animals each one. Group 1 received orally normal saline for 14 days, Group 2 was given orally 2.5% DMSO for 14 days, Group 3 received cefotaxime (200 mg/kg/day IM) for 14 days, Group 4 received with cefotaxime (200 mg/kg/day IM) and geranium oil (67 mg/kg b. w./day orally in DMSO) for 14 days, and Group 5 received geranium oil alone (67 mg/kg b. w./day orally in DMSO) for 14 days. Geranium oil significantly reduced cefotaxime-induced damage, evidenced by lower serum levels of liver enzymes (AST, ALT), renal markers (urea, creatinine), and other indicators (alkaline phosphatase, TNF-alpha, IL-1Beta, MAPK, nitric oxide, MDA). It also increased levels of protective tissue biomarkers such as NrF2, albumin, catalase, Beclin 1, and reduced glutathione (GSH). Histopathological and immunohistochemical analyses revealed significant protective effects in liver and renal tissues in rats treated with Geranium oil. These results suggest that Geranium oil is effective in mitigating cefotaxime-induced hepatotoxicity and renal toxicity.

## 1 Introduction

Cefotaxime is a broad-spectrum antibiotic that inhibits both aerobic and anaerobic bacteria, with Gram-negative bacteria being its primary target (Elkomy et al., 2020). As a result, it is used to treat a variety of infections, including typhoid fever, endocarditis, pneumonia, brain abscess, gonorrhoea, and meningitis. However, cefotaxime may cause several adverse effects, including eosinophilia and anaphylaxis, in addition to its toxic effects on the stomach, lung, liver, and kidney cells. (Salman and Hassoon, 2019). The use of injectable cephalosporins, such as cefotaxime, in the treatment of moderate to severe infections can sometimes result in drug-induced liver disease. This condition leads to elevated liver enzymes, hepatitis, or more severe liver damage, underscoring the importance of monitoring liver function during therapy (Al Sammak et al., 2021) and typical side effects of cefotaxime include rash, diarrhoea, and changes in renal and hepatic function test results (Sharma et al., 2024). Cefotaxime treatment may also cause detrimental changes in biochemical markers in addition to histological alterations in the liver and kidney (Roelofsen et al., 2023).

Exploring natural therapies as supplements or substitutes for traditional pharmaceutical interventions has gained popularity in recent years. Because of their various medicinal benefits and long history of usage in traditional medical systems, essential oils have drawn a lot of attention among these. Concentrated hydrophobic liquids with volatile fragrance molecules derived from plants are called essential oils. These oils are obtained via solvent extraction, distillation, or expression from a variety of plant parts, including leaves, flowers, stems, roots, bark, or fruits. Essential oils are utilised in aromatherapy, perfumery, cosmetics, and traditional medical procedures because of their distinctive scents (El-Sakhawy et al., 2023; Dagli et al., 2024). One of the therapeutic herbs with the highest antioxidant activity is rose-scented geranium (*Pelargonium graveolens* L’Hér.). Historically, geranium essential oil has been used to cure a variety of conditions, including cancer, heavy menstrual flows, inflammation, hemorrhoids, and dysentery (M’hamdi, et al., 2024). Kang et al. (2010) reported that essential oil-based mouth care, including geranium, lavender, tea tree, and peppermint oils, significantly improved oral comfort and health, as well as reduced *Candida albicans* colonization in terminal cancer patients compared to saline mouth care. Chaudhary et al. (2013) demonstrated that derivative of geranium (Geraniol), a natural monoterpene, shows strong chemopreventive effects in a skin cancer model. GOH reduced inflammation, oxidative stress, and tumor formation while inhibiting key cancer pathways and promoting apoptosis (Chaudhary et al., 2013), suggesting its potential as a cancer preventive agent. *Geranium incanum* leaf extract showed significant antidiarrheal effects, reducing fecal output, delaying diarrhea onset, and decreasing diarrheal episodes in a castor oil-induced model, comparable to loperamide (Amabeoku, 2009). Geranium oil, with its anti-inflammatory properties, has been shown to modulate microglial activity and key signaling pathways, potentially mitigating neuroinflammation in neurodegenerative diseases (Stojanović et al., 2024). Geranium essential oil is recognized in traditional Chinese medicine for its purported ability to support detoxification processes, thereby promoting balance in the body, and provides antioxidant protection (Ben Slima et al., 2013). *Pelargonium graveolens* essential oil demonstrated effective fungicidal activity, even against more resistant fungi like *Mucor mucedo* and *Aspergillus* species. Additionally, it exhibited strong dose-dependent antioxidant activity (Džamić et al., 2014). *Pelargonium graveolens* (geranium) essential oil (EOPG) showed potential in modulating physiological markers of mind-body balance. Inhalation of EOPG significantly lowers blood pressure and heart rate, indicating effects on autonomic regulation. Key compounds—linalool, citronellol, and geraniol—detected in the brain after exposure may underlie these cardioprotective effects, suggesting a mechanism by which EOPG supports autonomic stability and mind-body equilibrium (Masubuchi et al., 2019).

The p38 mitogen-activated protein kinase (MAPK) is an integral component of the MAPK cascade. This kinase is activated in response to oxidative stress, playing a crucial role in regulating the intracellular redox state (Gong et al., 2015). By adjusting the redox balance within cells, p38 MAPK helps to maintain cellular homeostasis and protect against oxidative damage (Lee et al., 2023). Nuclear factor erythroid 2-related factor 2 (Nrf2) is separated from Kelch-like ECH-associated protein 1 (Keap 1) and translocated into the nucleus in response to oxidative stress. It has been demonstrated that the p38-Nrf2 signalling pathway controls oxidative stress both *in vivo* and *in vitro*. Prior research revealed that HepG2 cells exposed to toxicants showed a considerable increase in p38 phosphorylation in addition to the formation of reactive oxygen species (ROS) (Ze et al., 2013; Fayez et al., 2024). In addition, the upregulation of Nrf2 in the liver of rats was a result of the oxidative damage triggered by toxic agents. However, it is unclear currently whether NF-κB and MAPK overactivation contributes to cefotaxime-induced oxidative liver injury (De Bock, 2014).

Based on the evidence provided above, the purpose of this study was to explore the hypothesis that the metabolism of cefotaxime, oxidative stress, and the overactivation of the MAPK and NF-κB signaling pathways are related to the liver damage caused by cefotaxime. Because of its high metabolic rate and low levels of protective compounds, the liver is a primary organ affected by oxidative stress, we assessed the hepatic toxicity in rats exposed to cefotaxime for 14 days and compared the effects of coadministration of geranium oil with cefotaxime.

## 2 Materials and methods

### 2.1 Animals

Male Wistar albino rats were sourced from the animal house at the National Organization for Drug Control and Research (NODCAR).

### 2.2 Drugs

Cefotaxime (dissolved in normal saline, 200 mg/kg/day). It was given by intramuscular injection for 14 days (Al Sammak et al., 2021). The Geranium oil was obtained from the National Research Center (NRC), Dokki, Egypt, and was extracted from the *P. graveolens* plant. The main components and their concentrations of this oil have been detailed in (Fahmy et al., 2023).

### 2.3 Experimental design and ethical approval

**FIGURE 1:**
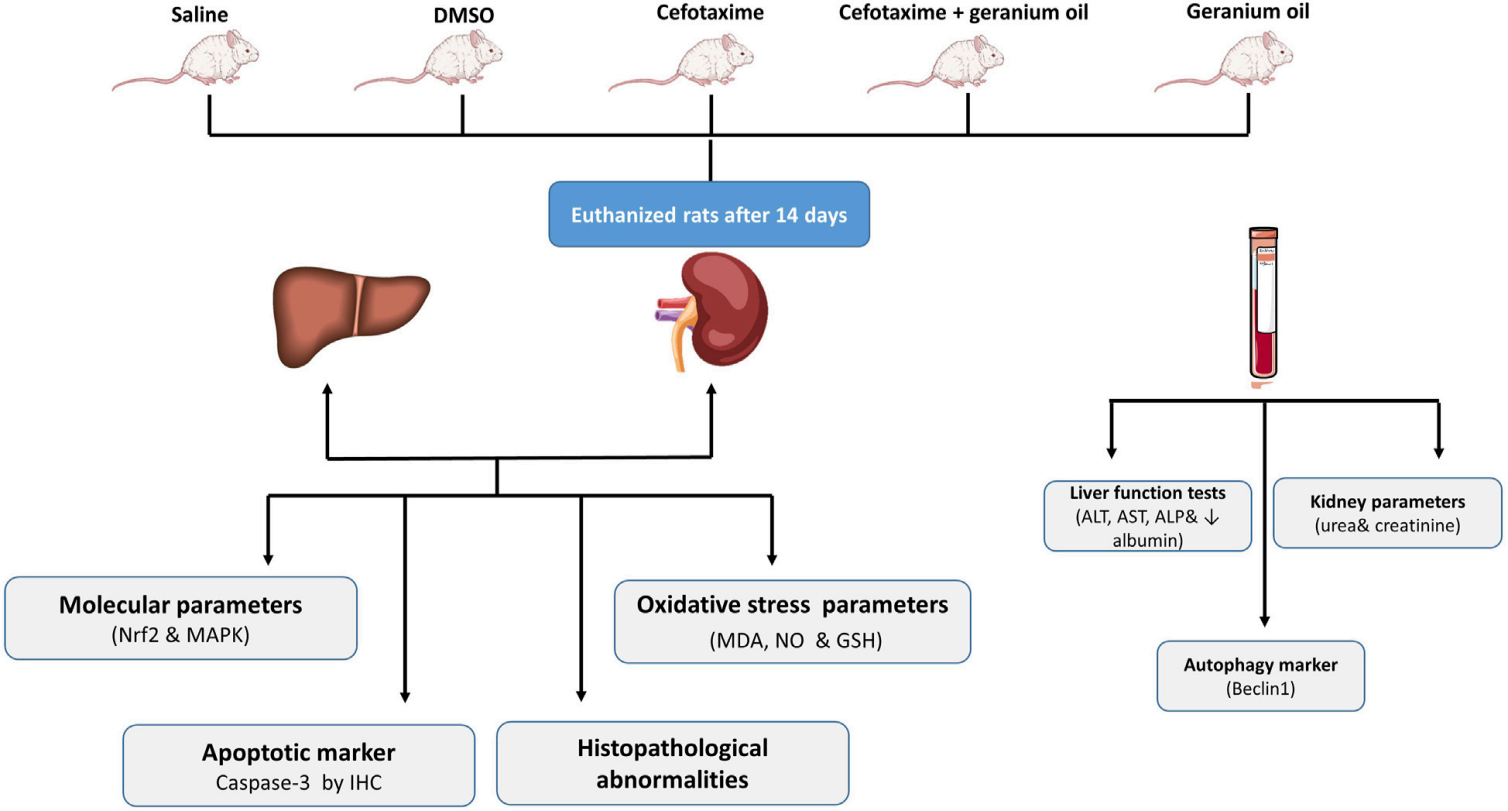
Schematic representation of the study methodology.

The current experimental study was conducted on male Wistar albino rats, aged 3–4 months and weighing between 170–200 g. The handling of the rats followed the protocols established by the NODCAR. They were selected from a pure strain to ensure a consistent and uniform genetic background. The animals always had free access to food and water and were kept in an environment maintained at 21°C–24°C with 40%–60% relative humidity and a 12-h light-dark cycle. To minimize stress, the animals were handled gently, avoiding squeezing, pressure, or rough maneuvers. All animal experimentation protocols were approved and supervised by the ethical committee of the National Organization of Drug Control and Research (NODCAR), in accordance with the organization’s guidelines. The approval reference number is NODCAR/II/39/2022. The study methodology is illustrated in Figure 1, which provides a schematic overview of the key steps involved in the research process.

Experimental Animals (30) Wistar albino male rats were randomly assigned into 5 groups of 6 rats in each group:

I. Control group (Negative group): Animals were received normal saline by oral gavage 14 days.
II. DMSO group: Animals were served as Controls received a DMSO 2.5% alone by oral gavage for 14 days (Kennedy-Feitosa et al., 2016).
III. Cefotaxime group (Positive group): Animals were received Cefotaxime (dissolved in normal saline, 200 mg/kg/day). It was given by intramuscular injection for 14 days (Al Sammak et al., 2021).
IV. Geranium Oil + Cefotaxime Group: Animals received geranium oil (67 mg/kg/day) dissolved in DMSO via oral gavage (Ben Slima et al., 2013), and cefotaxime (200 mg/kg/day) dissolved in normal saline via intramuscular injection for 14 days (Al Sammak et al., 2021).
V. Geranium oil group only: Animals were received Geranium oil dissolved in DMSO (67 mg/kg b. w/kg/day). It was given by oral gavage for 14 days (Ben Slima et al., 2013).

At the end of the experiment, 24 h after the last manipulation, the rats were anesthetized with an intraperitoneal injection of ketamine (90 mg/kg body weight) and xylazine (10 mg/kg body weight), both warmed to 37°C. Following anesthetization, the rats were euthanized, and blood samples were collected for serum separation. The liver and kidneys were immediately harvested and preserved at −80°C for biochemical analysis. A portion of the tissue was homogenized in protease/phosphatase-complemented lysis buffer (10% glycerol; 200 mM NaCl, 5 mM EDTA, 10 mM Tris, pH 7.4) for ELISA assays. The homogenate was centrifuged at 10,000 g for 15 min at 4°C, and the supernatant was stored at −80°C for further use. The organs from each group were preserved in 10% formalin-buffered saline for histology and immunohistochemistry. The animals that were used were then frozen until they were incineration.

### 2.4 Gene expression determination of MAPK&Nrf2 in liver and kidney tissue by RT-PCR

For RT-PCR gene expression analysis, about 30 mg of tissue was preserved in RNA lysis solution at −80°C until further processing. The expression of MAPK and Nrf2 genes was evaluated via real-time quantitative reverse transcription PCR (RT-PCR). Total RNA was extracted from the frozen samples using TRIzol^®^ reagent (Invitrogen, Sigma–Aldrich, St. Louis, MO) following standard procedures. This RNA was then converted into complementary DNA (cDNA) using SMARTScribe™ Reverse Transcriptase (Clontech Laboratories, Inc., a Takara Bio Company). RT-PCR was conducted with a Real-Time PCR v 7.9 System (DTlite, DNA Technology, LLC, Moscow, Russia) and SYBR^®^ Green PCR Master Mix (QIAGEN) in a 25 µL reaction volume. The cycling conditions included an initial step at 95°C for 15 s, followed by 40 cycles of 95°C for 15 s, 60°C for 15 s, and 72°C for 45 s. The primer sequences used are detailed in Table 1. Data were analyzed using ABI Prism sequence detection system software and quantified with PE Biosystems v17 Sequence Detection Software. Gene expression levels were determined using the comparative threshold cycle method, with all data normalized against the GAPDH gene as a reference control (Table 1).

**TABLE 1:** Gene Primer Sequences utilized in RT-PCR.

	Sequence	Accession number
MAPK	Forward: 5′CGAAATGACCGGCTACGTGG3′ Reverse: 5′CACTTCATCGTAGGTCAGGC3′	XM_017159206.1
Nrf2	Forward:5′CAAATCCCACCTTGAACACA 3′ Reverse:5′CGACTGACTAATGGCAGCAG 3′	XM_032903520.1
GAPDH	Forward: 5′GACAGTCAGCCGCATCTTCT3′ Reverse: 5′GCGCCCAATACGACCAAATC3′	XM_003819132.3

### 2.5 Oxidative stress and antioxidant parameters

The levels of reduced glutathione (GSH) and malondialdehyde (MDA) in the tissue were quantified spectrophotometrically according to the method outlined by Mihara and Uchiyama 1978. Catalase Activity (CAT) was determined spectrophotometrically according to the method of Aebi 1984, using the reagent kits purchased from Biodiagnostic company (Egypt). Nitric oxide (NO) levels were measured spectrophotometrically using the method described by Montgomery and Dymock 1961, with reagent kits obtained from Biodiagnostic Company (Egypt).

### 2.6 Liver and kidney function parameters

Aspartate aminotransferase (AST) and alanine aminotransferase (ALT) activities in serum were measured using reagent kits (Cat. nos. AL 1031, AS 1061) obtained from Bio diagnostics. For analyzing Aspartate (ALP), biochemical kits Spin React (Barcelona, Spain) were utilized (Salem et al., 2023). Colorimetric assay kits from Biomed Diagnostics (Cairo, Egypt) were utilized to measure blood urea, serum creatinine, serum albumin, and total protein levels (Khames, et al., 2019).

### 2.7 Measurement of (TNF-α), interleukin one beta (IL-1 β) and beclin 1

Rat TNF-α and IL-1β levels were measured in serum using enzyme-linked immunosorbent assay ELISA kits, following the manufacturer’s instructions provided by RayBiotech Inc. (Parkway Lane Suite, Norcross, GA) (Khames, et al., 2019). Beclin 1 levels were measured using an ELISA employing the sandwich technique, as provided by Sino Geneclon Biotech Co. Ltd. (Hangzhou, China) (Mohammed et al., 2020).

### 2.8 Histopathological examination and masson trichrome

Male rats were euthanized at the end of the trial, and tiny, fresh liver and kidney specimens were gathered and quickly preserved in 10% formalin solution for at least 24 h. The specimens underwent the standard paraffin embedding procedure, which involves dehydration in progressively higher grades of ethyl alcohol, clearing in various xylene changes, and embedding in various melted paraffin wax changes at 60°C. A microtome was used to cut paraffin blocks into 5-micron pieces. All tissue sections were examined under an Olympus BH-2 microscope for characterization of histopathological changes at scale bar 20 µm. The tissue sections were thick sections that were stained with Haematoxylin and Eosin (H.and E.) for histological evaluation of hepato-renal injury. Following an injection of cefotaxime and oil, the following parameters were selected to represent the degree of morphological damage to the liver: Congestion, Lymphatic infiltration, Necrosis, Hydropic degeneration and Pyknosis. And kidney: Lymphatic infiltration, Tubular degeneration, Fragmented glomeruli, and vacuolated glomeruli. These parameters were assessed on a scale ranging from absent or normal (−), mild (+), to moderate (++). Additionally, liver fibrosis was evaluated in Masson’s trichrome-stained liver sections. Coded samples were examined and scored under a light microscope in a blinded manner.

### 2.9 Immunohistopathological examination of Caspase-3

Sections of 4 μm thick were cut from various animal species, and immunohistochemistry was carried out. After deparaffinizing, rehydrating, and using H_2_O_2_ in methanol to inhibit endogenous peroxidase activity, sections were prepared. In a microwave, sections were pre-treated in citrate buffer (pH 6.0). Sections were treated with anti-caspase-3 antibodies (1:200) at room temperature. Sections were treated first with streptavidin peroxidase, then with biotinylated goat anti-polyvalent, and lastly with DAB plus chromogen. Hematoxylin was used as a counterstain on the slides. Under a light microscope, the slides were examined to determine the degree of cell immunopositivity.

### 2.10 Statistical analysis

The data were analyzed statistically using GraphPad Prism Software Inc., version 6.0. Results are presented as mean ± standard error of the mean (SEM). Statistical significance for biochemical parameters was determined with a threshold of *p* < 0.05. Analysis was conducted using one-way ANOVA, followed by a Tukey test for multiple comparisons.

## 3 Results

### 3.1 Effects of Cefotaxime and Geranium Oil on liver and renal function markers

In the present study, results showed that intramuscular injection of cefotaxime during the course of the experimental induced significant (*p* < 0.05) increase in AST, ALT and ALP serum activities as shown in Table 2. The table presents the effects of different treatments on serum activites of hepatic enzymes, specifically AST, ALT and ALP, which are markers of liver function and damage. In the control group, the activities of AST, ALT, and ALP were found to be 42.19 ± 0.89 IU/L, 29.88 ± 0.59 IU/L, and 59.25 ± 1.18 IU/L, respectively. These values represent normal liver enzyme activities in serum. For the DMSO group, which served as a vehicle control, enzyme activitites were similar to the control, with AST at 42.03 ± 0.66 IU/L, ALT at 31.57 ± 0.29 IU/L, and ALP at 64 ± 0.43 IU/L. In contrast, the group treated with cefotaxime exhibited a marked increase in liver enzyme activities: AST levels rose significantly to 201 ± 4.7 IU/L, ALT levels to 157.7 ± 6.64 IU/L, and ALP levels to 235.2 ± 5.75 IU/L. These elevations indicate substantial hepatotoxicity and liver damage caused by cefotaxime.

**TABLE 2:** Effects of cefotaxime and geranium oil on serum liver enzyme activities.

	AST (IU/L)	ALT (IU/L)	ALP (IU/L)
Control	42.19 ± 0.89	29.88 ± 0.59	59.25 ± 1.18
DMSO	42.03 ± 0.66	31.57 ± 0.29	64 ± 0.43
Cefotaxime	201 ± 4.7*	157.7 ± 6.64*	235.2 ± 5.75*
Cefotaxime + Geranium oil	83.1 ± 1.5#	69.58 ± 1.23#	99.34 ± 0.42#
Geranium oil	42.69 ± 0.99	34.21 ± 0.33	62.31 ± 1.4

The data are presented as mean ± SE (n = 6 rats/group). The symbols *Significant difference from the control group at *p* < 0.05. #Significantly different from the cefotaxime group at *p* < 0.05.

Comparing with cefotaxime group, liver enzyme activities decreased with the combination of cefotaxime and geranium oil. The AST, ALT, and ALP activities dropped to 83.1 ± 1.5 IU/L, 69.58 ± 1.23 IU/L, and 99.34 ± 0.42 IU/L, respectively. These decreases imply that geranium oil offers some defense against cefotaxime-induced liver damage. Enzyme activities for AST, ALT, and ALP were 42.69 ± 0.99 IU/L, 34.21 ± 0.33 IU/L, and 62.31 ± 1.4 IU/L for the geranium oil group alone. These results were in line with those of the control group, suggesting that geranium oil alone has no deleterious effects on liver function. Overall, these findings show that although cefotaxime causes severe liver damage, geranium oil successfully reduces hepatic damage and returns enzyme levels to normal.

As showed in Table 3, Creatinine, albumin, and urea serum levels were normal in the control and DMSO groups. Renal impairment was evidenced by a considerable increase in serum creatinine (2.02 ± 0.06 mg/dL) and urea (87.25 ± 1.2 mg/dL) levels during cefotaxime treatment, and a decrease in serum albumin levels (2.8 ± 0.03 g/dL). After receiving cefotaxime + geranium oil treatment, serum levels of albumin (3.45 ± 0.02 g/dL) and urea (49.88 ± 0.59 mg/dL) were restored, indicating a potential protective effect against kidney impairment. Creatinine, albumin, or urea levels were not signficantly different from the control group while using geranium oil alone. This research suggests that kidney damage is induced by cefotaxime, but that these effects can be prevented by geranium oil.

**TABLE 3:** Effects of cefotaxime and geranium oil on renal function markers.

	Creatinine (mg/dL)	Albumin (g/dL)	Urea (mg/dL)
Control	0.44 ± 0.01	3.8 ± 0.028	31.03 ± 0.74
DMSO	0.49 ± 0.01	3.77 ± 0.05	34.51 ± 0.34
Cefotaxime	2.02 ± 0.06*	2.8 ± 0.03*	87.25 ± 1.2*
Cefotaxime + Geranium oil	1.03 ± 0.02#	3.45 ± 0.02#	49.88 ± 0.59#
Geranium oil	0.44 ± 0.01	3.79 ± 0.03	33.81 ± 0.43

The data are presented as mean ± SE (n = 6 rats/group). The symbols: The data are presented as mean ± SE (n = 6 rats/group). The symbols *Significant difference from the control group at *p* < 0.05. #Significantly different from the cefotaxime group at *p* < 0.05.

### 3.2 Effect of geranium oil on cefotaxime-induced oxidative stress markers, and biochemical parameters in liver and kidney tissues

As showed in Table 4, catalase activity was significantly lower (8.64 ± 0.11 units) after receiving cefotaxime therapy than in the DMSO (14.34 ± 0.18) and control groups (14.38 ± 0.38 units) groups. Catalase activity was higher with geranium oil (10.96 ± 0.4) as compared to the cefotaxime group. Nitric oxide (NO) levels were significantly higher after cefotaxime treatment (127.7 ± 4.3) than in the control (35.86 ± 6.05) and DMSO groups (44.52 ± 0.54) groups. When compared to the cefotaxime group, geranium oil treatment resulted in a considerable reduction of nitric oxide levels (76.46 ± 1.77). The cefotaxime group had significantly higher MDA levels (74.33 ± 0.59 units) than the DMSO group (31.63 ± 0.5) and control group (28.81 ± 1.1). Compared to the cefotaxime group, MDA levels were significantly lower (52.86 ± 0.85) after geranium oil treatment. GSH levels were significantly lower in the cefotaxime group (64.34 ± 0.27 units) than in the DMSO group (27.31 ± 0.59) and control group (25.86 ± 0.54). GSH levels were significantly higher (40.41 ± 0.56) after geranium oil treatment than in the cefotaxime group.

**TABLE 4:** Effect of geranium oil on cefotaxime-induced oxidative stress and biochemical parameters in liver tissues.

Group	Catalase (U/mL)	Nitric oxide µ mol/L	MDA nmol/L	GSH µmole/g tissue
Control	35.86 ± 6.05	28.81 ± 1.1	25.86 ± 0.54	14.38 ± 0.38
DMSO	44.52 ± 0.54	31.63 ± 0.5	27.31 ± 0.59	14.34 ± 0.18
Cefotaxime	127.7 ± 4.3*	74.33 ± 0.59*	64.34 ± 0.27*	8.64 ± 0.11*
Cefotaxime + Geranium oil	76.46 ± 1.77#	52.86 ± 0.85#	40.41 ± 0.56#	10.96 ± 0.4#
Geranium oil	41.40 ± 45.28	30.8 ± 0.67	26.35 ± 0.36	14.38 ± 0.38

The data are presented as mean ± SE (n = 6 rats/group). The symbols: The data are presented as mean ± SE (n = 6 rats/group). The symbols *Significant difference from the control group at *p* < 0.05. #Significantly different from the cefo. group at *p* < 0.05.

As showed in Table 5, cefotaxime therapy raised nitric oxide levels (78.04 ± 2.89 units) compared to the control (31.08 ± 1.35) and DMSO groups (32.64 ± 0.75), which was similar to liver tissue. When compared to the cefotaxime group, geranium oil administration resulted in a considerable reduction of nitric oxide levels (51.44 ± 0.3). Compared to the control (27.07 ± 0.56) and DMSO groups (28.44 ± 0.61) groups, the MDA levels were considerably higher in the cefotaxime group (69.93 ± 1.48 units). MDA levels were substantially lower (47.18 ± 0.46) in the geranium oil group compared to the cefotaxime group. Compared to the control group (14.5 ± 0.1) and DMSO group (14.26 ± 0.03), cefotaxime significantly reduced GSH levels (7.86 ± 0.104 units). GSH levels were higher in the geranium oil group (10.57 ± 0.2) than in the cefotaxime group. The cefotaxime group (8.64 ± 0.11) had significantly lower catalase activity than the DMSO (14.34 ± 0.18) and control groups (14.38 ± 0.38). Catalase activity was higher with geranium oil (10.96 ± 0.4) as compared to the cefotaxime group.

**TABLE 5:** Effect of geranium oil on cefotaxime-induced oxidative stress and biochemical parameters in kidney tissues.

Group	Nitric oxide µ mol/L	MDA nmol/L	GSH µmole/g tissue
Control	31.08 ± 1.35	27.07 ± 0.56	14.5 ± 0.1
DMSO	32.64 ± 0.75	28.44 ± 0.61	14.26 ± 0.03
Cefotaxime	78.04 ± 2.89*	69.93 ± 1.48*	7.86 ± 0.104*
Cefotaxime + Geranium oil	51.44 ± 0.3#	47.18 ± 0.46#	10.57 ± 0.2#
Geranium oil	30.38 ± 0.92	29.6 ± 0.8	14.08 ± 0.19

The data are presented as mean ± SE (n = 6 rats/group). The symbols: The data are presented as mean ± SE (n = 6 rats/group). The symbols *Significant difference from the control group at *p* < 0.05. #Significantly different from the cefo. group at *p* < 0.05.

### 3.3 Effects of Cefotaxime and Geranium Oil on inflammation markers

**FIGURE 2:**
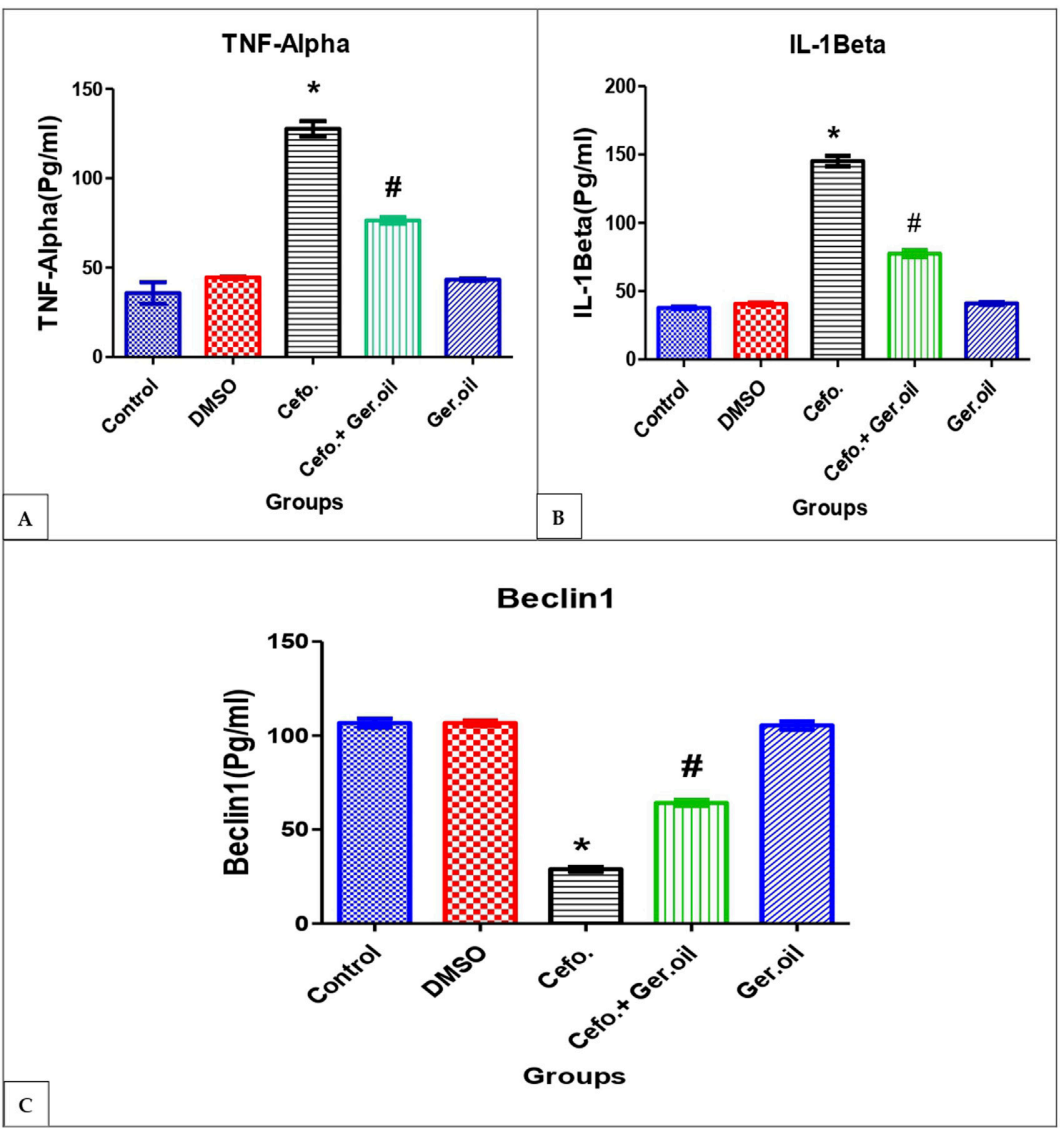
Effects of Cefotaxime and Geranium Oil on inflammation Markers, TNF- α **(A)**, IL-Beta **(B)** and Beclin-1 **(C)**. The data are presented as mean ± SE (n=6 rats/group). The symbols *Significant difference from the control group at p < 0.05. #Significantly different from the cefotaxime group at p < 0.05.

Exposure to cefotaxime resulted in a significant increase in TNF-α levels with an average of 127.65 compared to 41.84 in the control group, indicating an inflammatory response. Treatment with geranium oil in combination with cefotaxime decreased TNF-α levels to an average of 76.46, suggesting a potential protective or anti-inflammatory effect of geranium oil (Figure 2A). Similarly, IL-1 Beta levels in the Cefotaxime group increased to an average of about 145.53, while in the Cefotaxime + Geranium oil group they decreased to an average of 77.78, indicating that Geranium oil significantly reduced the cefotaxime-induced elevation of IL-1 Beta. This increase indicates a marked inflammatory response (Figure 2B). In the cefotaxime group, levels of the autophagy marker Beclin 1 were considerably reduced, falling to an average of 28.95 from the control average of 106.03. This suggests that cefotaxime may block autophagy. But Beclin 1 levels rose to an average of 64.57 when Geranium oil (Cefotaxime + Geranium) was present, suggesting that Geranium oil may help restore autophagic function to some extent (Figure 2C).

In the cefotaxime-treated group, there was a significant increase in pro-inflammatory markers compared to the control (*p* < 0.05, TNF-α; *p* < 0.05, for IL-1β), suggesting an inflammatory response. Co-administration with Geranium oil significantly reduced these elevated levels, as indicated by TNF-α values dropping from 127.65 ± 1.32 to 41.84 ± 4.353 (*p* < 0.05) and IL-1β levels from 145.53 ± 1.99 to 77.78 ± 6.241 (*p* < 0.05). Furthermore, Beclin one levels, which were reduced in the cefotaxime group (*p* < 0.05), were partially restored upon treatment with Geranium oil (64.57 ± 3.733, *p* < 0.05), suggesting a protective effect on autophagy pathways.

### 3.4 Effects of Cefotaxime and Geranium Oil on gene expression of MAPK and Nrf2 in liver and kidney

**FIGURE 3:**
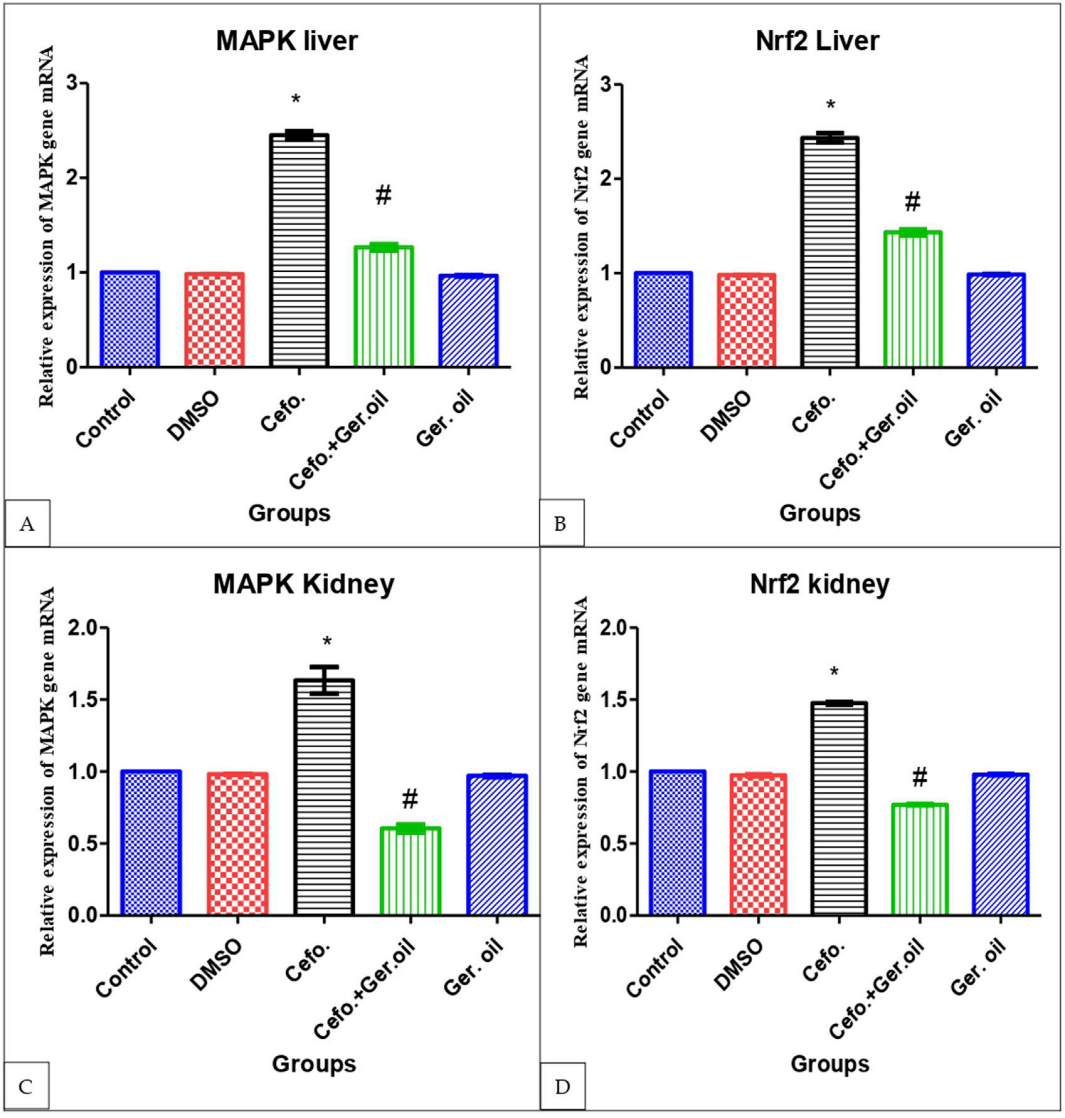
Effects of Cefotaxime and Geranium Oil on gene expression of MAPK **(A)** and Nrf2 **(B)** in liver and MAPK **(C)** and Nrf2 **(D)** in kidney. The data are presented as mean ± SE (n = 6 rats/group). The symbols: The data are presented as mean ± SE (n = 6 rats/group). The symbols *Significant difference from the control group at *p* < 0.05. #Significantly different from the cefo. group at *p* < 0.05.

MAPKs are activated through phosphorylation and subsequently stimulate the downstream transcription factor NF-κB. Our findings indicate that cefotaxime treatment in rats led to increased phosphorylation levels of p-38 MAPK. However, geranium oil treatment significantly decreased the phosphorylated protein levels of p-38 MAPKs, indicating the geranium oil’s protective effect in the cefotaxime-induced toxicity on rats as shown in Figures 3A, C. On the other hand, our findings showed that geranium oil treatment exert inhibitory effect on Nrf2 after its elevation by cefotaxime treatment as shown in Figures 3B, D.

### 3.5 Histopathology results of H &E staining method

**FIGURE 4:**
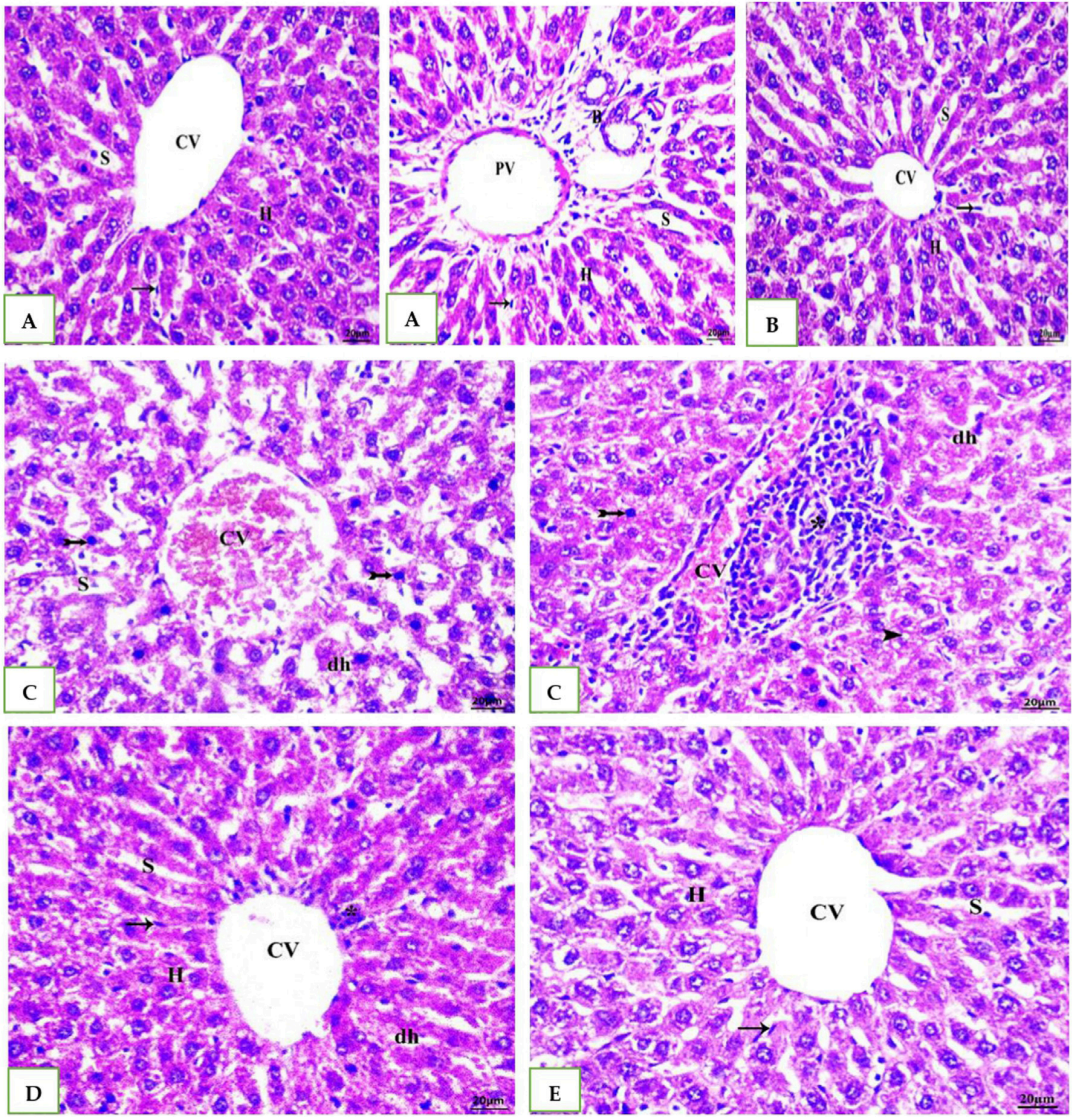
**(A, B, E)**: photomicrographs of section in liver of Normal control, DMSO and Geranium oil alone treated rats. Histological examination revealed classical hepatic lobules with hepatocyte cords radiating and interconnecting from a central vein. Hepatocytes exhibited a polyhedral shape, with rounded vesicular nuclei and acidophilic, finely granular cytoplasm. Kupffer cells were identified (arrow). Hepatic sinusoids separated the hepatocyte cords. A distinct portal triad, composed of a portal vein branch and bile duct, was evident. While **(C)** Cefotaxime positive group showing liver injury as dh = degenerated hepatocyte, N = necrosis, Asterisk = lymphatic infiltration and Bifid arrow = pyknotic. But in **(D)** Geranium oil + cefotaxime group induce marked improvement in the histological damage compared with cefotaxime alone treated group (H&E scale bar 20 µm).

As shown Table 6 and in A photomicrograph of a liver sample stained with Hematoxylin and Eosin Figure 4: The control rat’s anatomy (Figure 4A): was normal, with no histological abnormalities, as well as a typical central vein and hepatocyte arrangement. Hepatocyte nuclei were seen as dark red objects inside the cells, with the cytoplasm dyed red. The sinusoids and the typical portal region, along with comparable hepatic strands that go from the margin of the lobule to the central vein. In G2 DMSO group (Figure 4B): standard composition of hepatic tissue, but there were numerous congested blood vessels. Moreover, G3 (Cefotaxime group): In liver sections (Figure 4C): there was altered lobular form and nuclear deterioration in some locations, as well as disarray of normal hepatic cells, necrosis, and hydropic degeneration. Enlargement and moderate congestion of the hepatic central vein were discovered. In the portal area, moderate lymphocyte infiltration was seen. While in G4 (Geranium oil + cefotaxime group): Hepatocytes and portal components (Figure 4D): appeared to be in good health. The region is infiltrated by small mononuclear cells. Necrotic changes were not detected. It was possible to see a few clogged blood vessels. There was mild hydropic degeneration. Few numbers of hepatic cells revealed pyknotic feature. As expected in G5 (Geranium oil only group). Hepatic architecture appeared normal, with hepatic cords properly aligned around sinusoids. There was no evidence of mononuclear cell invasion. There were no alterations in fatty degeneration noted. The blood vessels appeared to be normal as in Figure 4E.

**TABLE 6:** Semi-quantitative analysis of histology of Liver of rats.

	Congestion	Lymphatic infiltration	Necrosis	Hydropic degeneration	Pyknosis
G1 Control Normal	-	-	-	-	-
G2 DMSO	++	-	-	-	-
G3 Cefotaxime	++	++	+	++	++
G4 Geranium oil + Cefotaxime	+	+	-	+	+
G5 Geranium oil	-	-	-	-	-

(−) indicates normal, (+) indicates mild, (++) indicates moderate.

**FIGURE 5:**
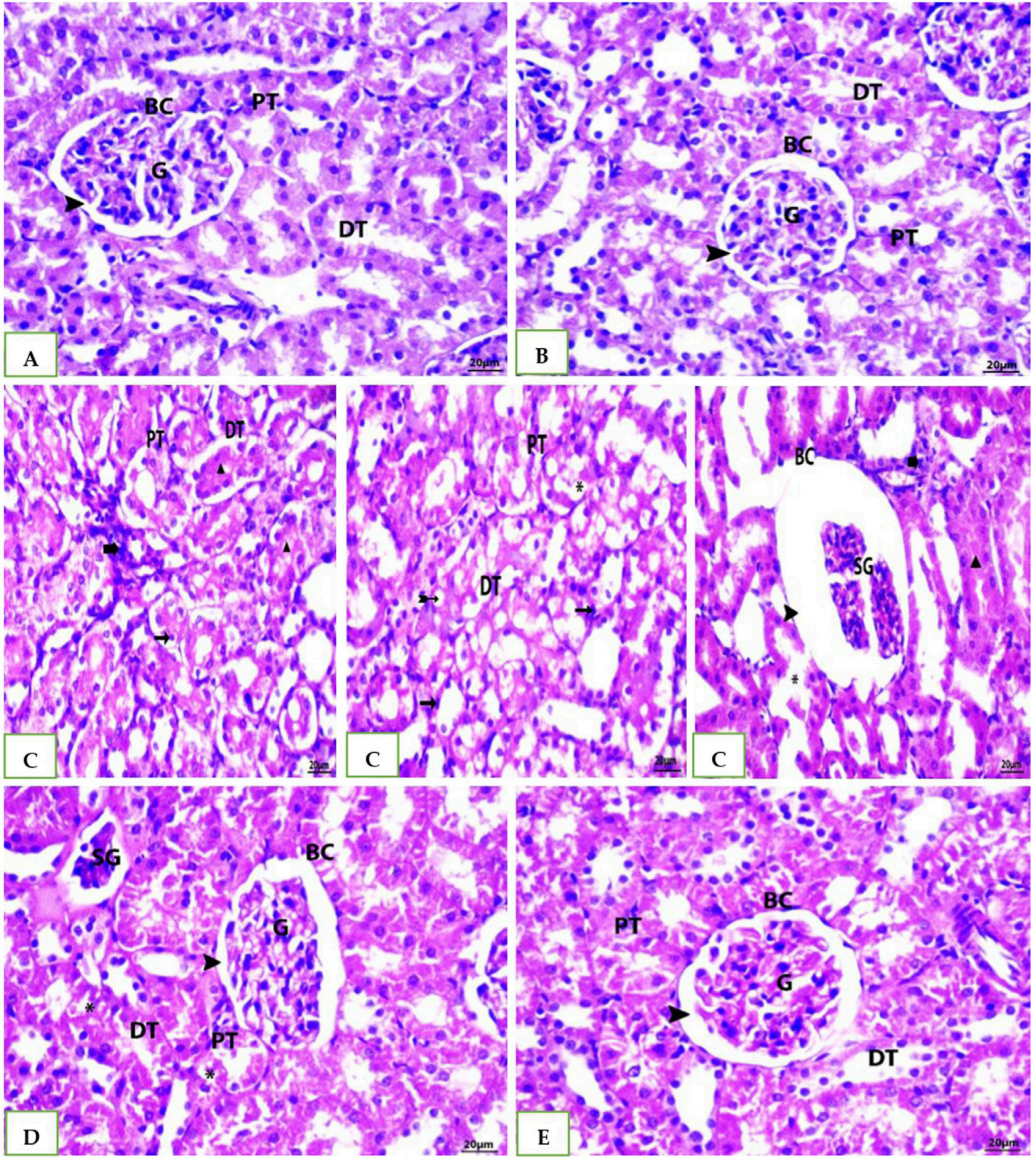
Photomicrographs of rat kidney (Scale bar 20 µm) from: **(A)** control group **(B)** DMSO group and **(E)** The geranium oil-only group exhibited normal renal architecture. In contrast **(C)** the cefotaxime group displayed severe renal damage characterized by extensive necrosis, tubular dilation, vacuolar degeneration, epithelial cell shedding, and intraluminal cast formation primarily within the proximal tubules; **(D)** Geranium oil + cefotaxime group displaying marked improvement in the histological picture. BC = Bowman’s capsule, G = glomeruli, DG = degenerated glomeruli, E.G., = enlarged glomeruli, SG = shrunken glomeruli, CG = congested glomeruli, PT = proximal tubule, DT = distal tubule, Head arrow = capsular space, Star = congestion,Arrow = pyknotic nuclei,Bold arrow = lymphatic infiltration, Asterisk = degenerated tubule, Triangle = luminal cast, Bifid arrow = vacuolation.

The H&E staining results represented in Table 7 and Figure 5. G1: Renal (Malpighian) corpuscles with glomerular capillaries, Bowman’s capsules, and urine space had a typical histological appearance in the renal cortex. Proximal convoluted tubules had a small lumen lined with cubical cells at the base with round nuclei. Distal convoluted tubules had a wide lumen, which was surrounded by simple cubical cells with sphere nuclei in the center or at the apex. The rats in the G2and5 had renal cortical structures that were similar to those in the control group. The group treated with cefotaxime exhibited some degree of degeneration; glomerular asymmetry in size and structure, with some glomeruli shrinking in size due to atrophied glomerular tuft and cystic appearance. Some vacuolated, fragmented and congested glomeruli were seen. Cell nuclei showed full or partial destruction in the proximal and distal convoluted tubules. Among the damaged tubules and renal corpuscles, there was mononuclear cell invasion. Renal tubules with disturbed epithelial linings and desquamation were seen. Some renal tubules showed luminal cast. G4 (Figure 5D) Geranium oil + cefotaxime group: some tubular epithelial cells in renal convoluted tubules appeared to have entire cytoplasm, whereas others had mild vacuolation. The glomeruli with normal and standard size inside Bowman’s capsule. There was no evidence of lymphatic invasion. The most of renal glomeruli and tubules appeared normal.

**TABLE 7:** Semi-quantitative analysis of histology of kidney of rats.

	Lymphatic infiltration	Vacuolated, fragmented, shrinkage glomeruli	Tubular degeneration
G1 Control Normal	-	-	-
G2 DMSO	-	-	-
G3 Cefotaxime	+	++	++
G4 Geranium oil + Cefotaxime	-	+	+
G5 Geranium oil	-	-	-

(−) indicates normal, (+) indicates mild, (++) indicates moderate.

### 3.6 The Masson’s trichrome staining results of liver

**FIGURE 6:**
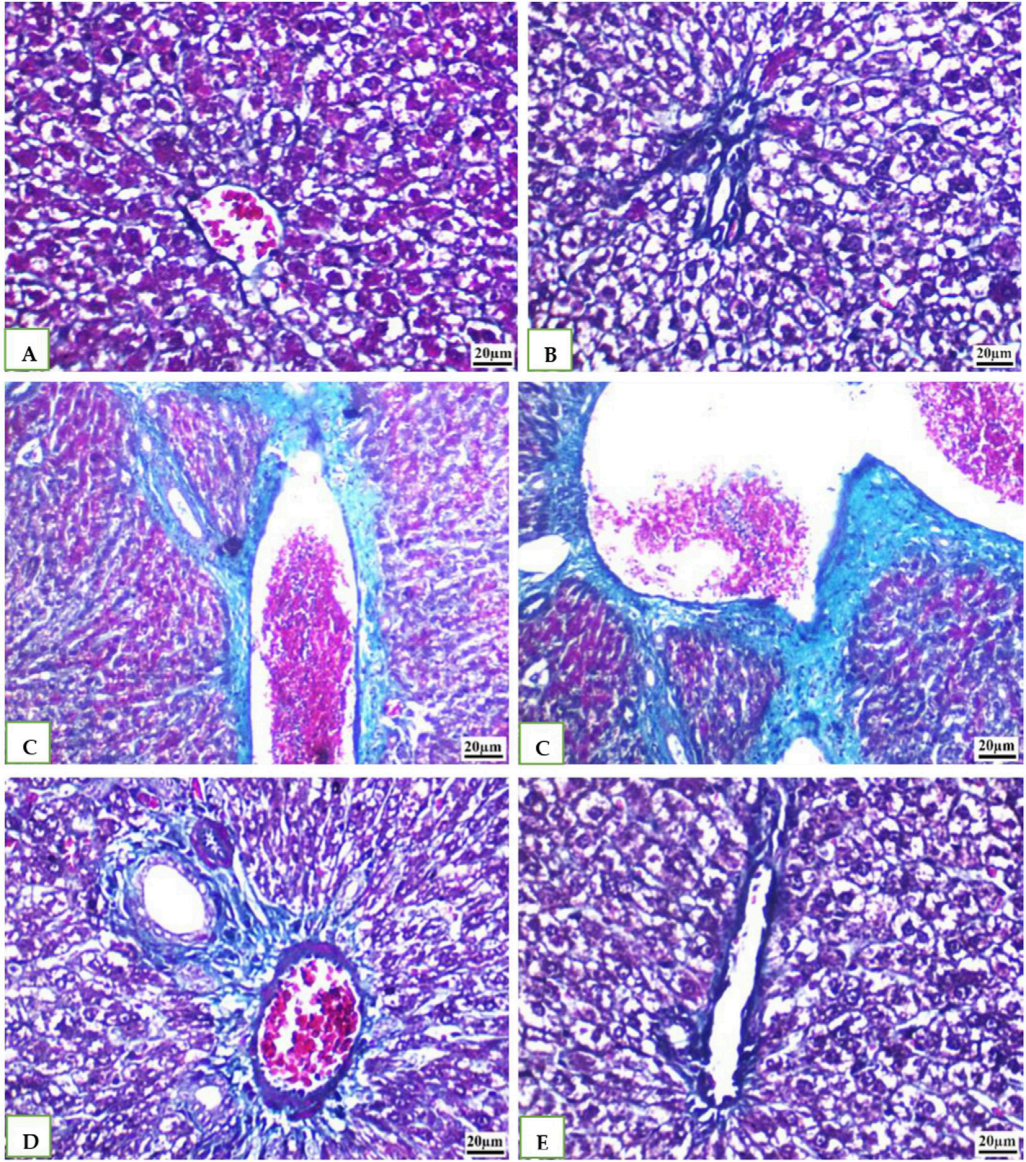
**(A, B, E)**: photomicrographs of section in liver of Normal control, DMSO and Geranium oil alone treated rats. **(C)** Cefotaxime positive group **(D)** Geranium oil + cefotaxime group (Masson’s trichrome staining scale bar 20 µm).

Histological examination of tissue samples from groups G1, G2, and G5 (Figures 6A, B, E) revealed delicate collagen fibers within the central vein walls of hepatic lobules. Minimal collagen deposition was also observed in the interlobular septa and surrounding the portal vein. In the cefotaxime group (Figure 6C), a marked increase in collagen fiber deposition was observed, leading to the formation of a fibrous septum within the hepatic lobule. Fiber accumulation intensified in the portal region, interacting with nearby septa, and wrapping around each other. Compared to the model group, the G4 (Figure 6D) group treated with both geranium oil and cefotaxime exhibited a significant reduction in collagenous fibrous tissue. Fibrosis was markedly decreased, with only a minimal amount of thin, blue-stained fibers observed around blood vessels.

### 3.7 Caspase 3 immunohistochemical staining (IHC)

**FIGURE 7:**
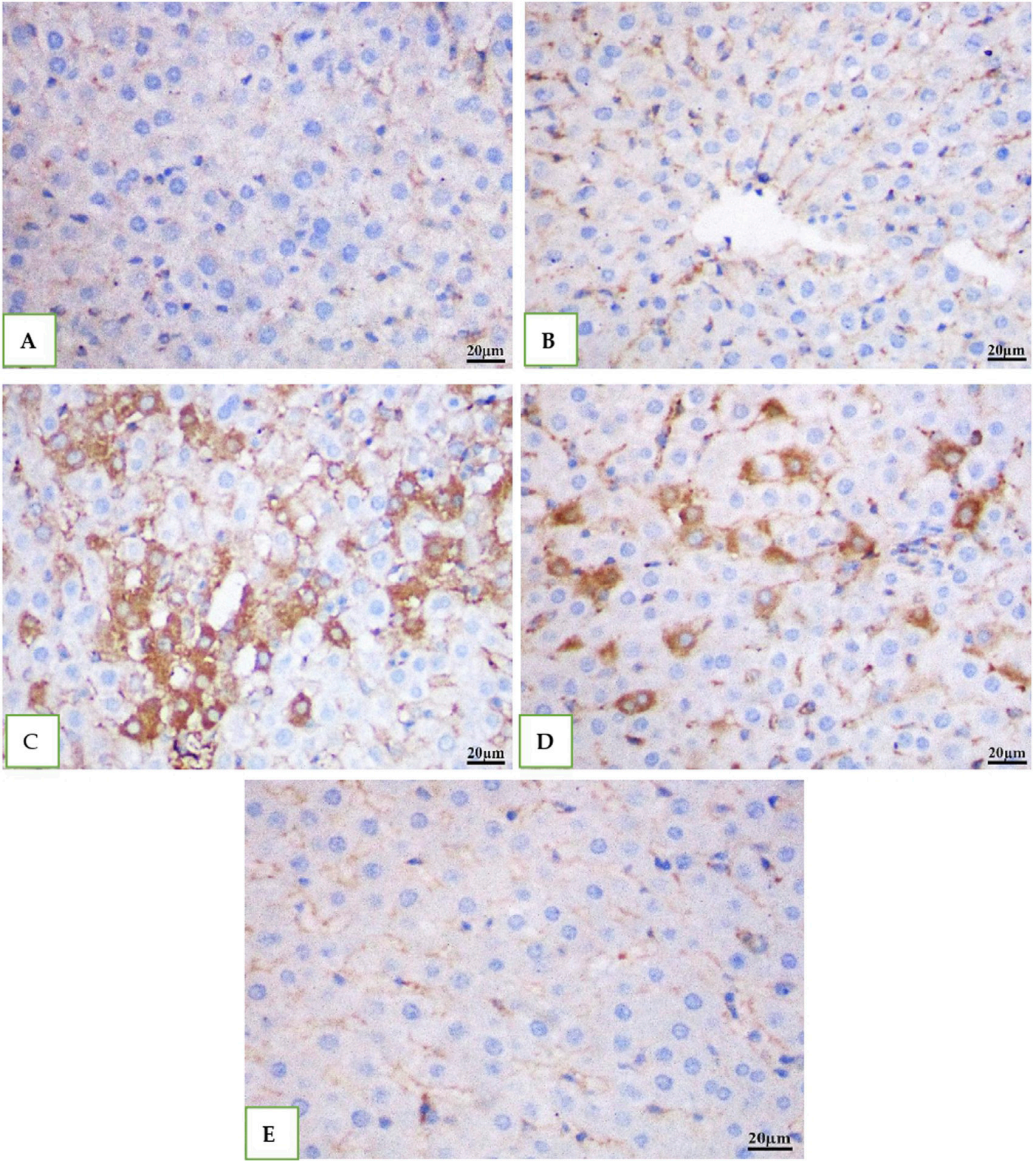
Immunohistochemical staining for caspase-3 in rat liver tissue (scale bar: 20 µm). Groups **(A)** (normal control), **(B)** (BMSO), and **(E)** (geranium oil) exhibited no caspase-3 expression. In contrast, the cefotaxime group **(C)** showed a marked increase in caspase-3 immunoreactivity within the cytoplasm of proximal tubular cells. Co-treatment with geranium oil and cefotaxime [group **(D)**] significantly reduced caspase-3 immunostaining compared to the cefotaxime group. Brown coloration indicates positive caspase-3 staining.

IHC was used to examine the expression of the caspase −3 in all the groups' rat livers. While DMSO and Geranium oil had no effect on caspase −3 levels (Figures 7B, E), the cefotaxime group showed a significant rise in caspase −3 positive levels (Figure 7C), which was dramatically reduced by geranium oil administration (Figure 7D).

**FIGURE 8:**
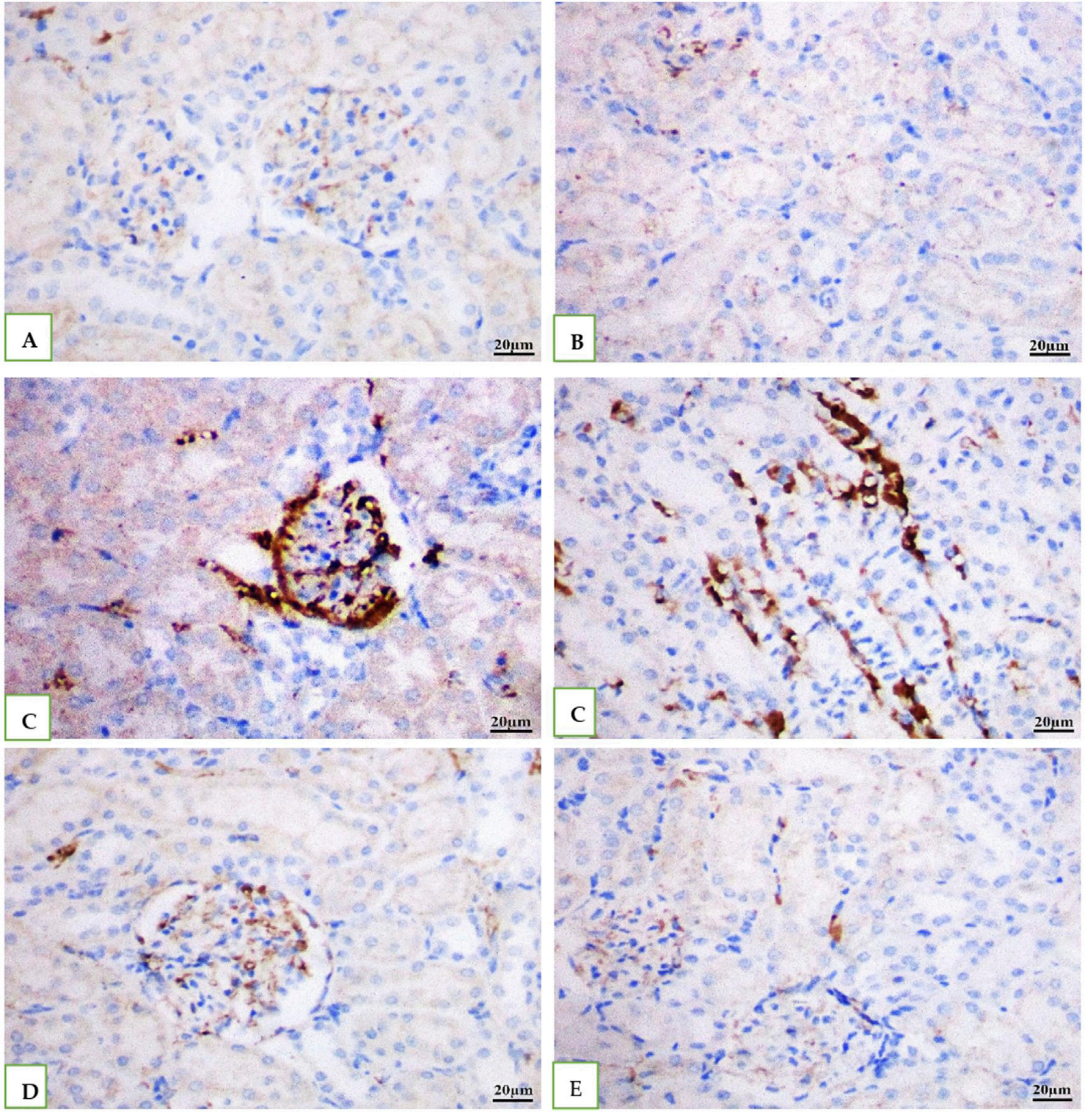
Depicts immunohistochemical staining for caspase-3 in rat kidney tissue (scale bar: 20 µm). Groups **(A)** (normal control), **(B)** (BMSO), and **(E)** (geranium oil) showed no caspase-3 expression. In contrast, the cefotaxime group **(C)** displayed a significant increase in caspase-3 immunoreactivity within the cytoplasm of proximal tubular cells. Co-treatment with geranium oil and cefotaxime [group **(D)**] resulted in a marked reduction of caspase-3 immunostaining compared to the cefotaxime group. Brown coloration denotes positive caspase-3 staining.

IHC was used to examine the levels of caspase −3 in all the groups' rat kidney. While Vehicle control DMSO and geranium oil had no effect on caspase −3 levels (Figures 8A, B, E), the cefotaxime injected group showed a significant rise in caspase −3 positive levels (Figure 8C), which was dramatically reduced by geranium oil treatment (Figure 8D).

## 4 Discussion

Geranium oil is well-known for its strong anti-inflammatory and liver-protective effects, which help in neutralizing damaging free radicals. The current study aimed to explore the molecular mechanisms by which geranium oil exerts protective effects on the liver and kidneys against damage induced by cefotaxime in rats. Specifically, the research investigated how geranium oil influences the MAPKs/NF-κB and AMPK/Nrf2 signaling pathways order to understand how these interactions contribute to anti-oxidative, anti-inflammatory, and protective effects on the liver and kidneys under cefotaxime-induced toxicity.

Cefotaxime was administered at a dose of 200 mg/kg/day via intramuscular injection for a period of 14 days. This treatment resulted in a remarkable increase in serum levels of AST, ALT, and ALP, signaling liver injury. The observed increase in these enzyme activities suggests hepatocellular damage, which includes fatty degeneration, fibrosis, and compromised liver function, leading to the release of intracellular enzymes into the bloodstream. Elevated levels of AST, ALT, and ALP serve as key markers of damage to hepatocyte membranes and cellular disruption. These findings are consistent with previous studies (El-Maddawy and Bogzil, 2015; Soliman, 2015). Cefotaxime also caused a notable decrease in serum albumin levels, which may be attributed to liver fibrosis. This condition likely results in a reduction in the number of hepatocytes, ultimately impairing protein synthesis. On the other hand, this albumin decrease was abolished after co-administration of geranium oil and cefotaxim. Concering kidney, cefotaxime significantly increased serum levels of creatinine and urea. These nephrotoxic effects of cefotaxime are coincides with that reported by Soliman 2015. However, oral administration of geranium oil led to a significant reduction in the elevated levels of serum creatinine and urea. Thus, geranium oil could protect against liver and kidney damage and enhance their function. These improvment is in accordance with the findings of Necib et al., 2014.

Cells have various antioxidant compounds and enzymes that help prevent and repair oxidative stress damage. For instance, the enzymatic antioxidant catalase (CAT) converts hydrogen peroxide (H₂O₂) into water and oxygen (Weydert and Cullen, 2010). Glutathione (GSH) acts as a cofactor for various enzymes and is involved in detoxifying hydrogen peroxide and lipid peroxides through its role with glutathione peroxidase. It also helps protect cells from apoptosis by interacting with pro-apoptotic signaling pathways, thereby balancing the effects of oxidative stress markers and antioxidants (Birben et al., 2012). In the present study, cefotaxime administration decreased GSH levels and increased MDA, NO, and CAT activity. The elevated MDA and NO levels are indicative of oxidative stress and lipid peroxidation, resulting from increased free radical generation. Excessive production of free radicals leads to increased lipid peroxidation (MDA) by damaging unsaturated fatty acids in cell membranes. This observation is consistent with findings by Abd El Fadil et al. (2018). Notably, our study found that administering geranium oil for 14 days alongside cefotaxime protected renal and hepatic tissues from oxidative stress. This protection was achieved by enhancing antioxidant defenses, as evidenced by increased GSH levels and decreased MDA, NO, and CAT activity, which is regulated by the Nrf2 signaling pathway. Nrf2 activates target genes encoding defense enzymes like HO-1 and GSH synthase/peroxidase, mitigating oxidative stress by maintaining cellular redox equilibrium (Iranshahy et al., 2018).

These findings align with previous studies (Singh et al., 2012 and Kasote et al., 2015). Geranium essential oil’s antioxidant properties arise from its ability to neutralize free radicals. It achieves this by intercepting reactive oxygen species and preventing them from damaging cellular components (Boukhris et al., 2012). The antioxidant effects of geranium essential oil can largely be credited to its monoterpene components, particularly geraniol and β-citronellol, which are predominant in its chemical makeup. Lu and Foo 2001; Nivitabishekam et al., 2009 reported that natural antioxidant often work synergistically, combining their effects to produce a comprehensive array of antioxidant properties. This synergistic action contributes to a robust defense system against free radicals, enhancing the overall effectiveness of the antioxidant response (Brito et al., 2012 and Lohani et al., 2018).

Inflammation, a complex biological response to oxidative injury or infection, is orchestrated by the immune system to eliminate harmful stimuli and initiate tissue repair. While essential for host defense, dysregulated inflammation can contribute to various pathologies (Xiao, 2017). Key inflammatory mediators, including TNF-α, IL-1β, COX-2, and NO, play pivotal roles in this process. Anti-inflammatory therapies often target these mediators to alleviate inflammation and its associated diseases (Fayez et al., 2024). Cefotaxime, in line with previous studies, induced an inflammatory response characterized by increased expression of TNF-α and IL-1β in this investigation. While, geraniol therapy markedly reduced the rise in serum activities of TNF-α and IL-1β induced by cefotaxime. These findings are in cosistent with early studies (Marcuzzi et al., 2011; Chaudhary et al., 2013). Where, geraniol lowers the levels of inflammatory markers *in vivo* and *in vitro* (Marcuzzi et al., 2011). Furthermore, geranio prevented the development of skin tumors through its anti-inflammatory effect (Chaudhary et al., 2013).

The MAPK signaling pathway regulates cellular processes, including proliferation, differentiation, apoptosis, stress responses and inflammation (Kim and Choi, 2015). Various stimuli, including inflammatory cytokines and reactive oxygen species, can activate the MAPK pathway, leading to the activation of transcription factors such as NF-κB and subsequent modulation of gene expression and cellular responses. Our findings show that liver and kidney p38 MAPK and NF-κB activation was caused by cefotaxime exposure, but this response was suppressed by geranium oil treatment. Notably, lower levels of pro-inflammatory mediators such TNF-α, IL-1β, COX-2, and iNOS as well as an inhibition of NF-κB signalling were linked to the geranium-mediated suppression of MAPK activity. These results imply that geranium oil interferes with the MAPK/NF-κB signalling axis to mediate its anti-inflammatory actions (Wang et al., 2016; Lee et al., 2023). Our findings point out that geranium oil treatment exert inhibitory effect on Nrf2 after its elevation by cefotaxime treatment; thus, the roles of MAPK and Nrf2 signaling pathway may be cell specific or rely on the biological activity of different antioxidants as explained by Li et al., 2024.

Autophagy, a cellular stress response, is implicated in various diseases, including acute kidney injury (AKI). In AKI, autophagy is activated in proximal tubules and plays a protective role, as demonstrated by pharmacological and genetic studies. While autophagy is essential for tubular cell proliferation and repair, it can also contribute to renal fibrosis by inducing cell atrophy and breakdown or mitigating fibrosis by degrading excess collagen (He et al., 2014). Beclin-1, a core component of Class III Phosphatidylinositol 3-Kinase (PI3K) complexes, is essential for autophagy and membrane trafficking, apoptosis, and cell proliferation signaling pathways (Kang et al., 2011; Tran et al., 2021).

In the current study, Cefotaxime elevated caspase-3 levels in both liver and kidney tissues, aligning with the findings of El-Emam et al., 2020. Conversely, geranium treatment decreased caspase-3 levels, suggesting its ability to reduce apoptosis by mitigating mitochondrial damage. This outcome corresponds with the results of previous research (Younis et al., 2021; Danış et al., 2023).

Histopathological analyses of liver and kidney tissues from animals receiving cefotaxime supported the results. These tissues showed signs of significant damage, including an influx of neutrophils and the presence of many apoptotic bodies, along with cell death (necrosis) in both organs. These findings are consistent with earlier research (Soliman, 2015; Jazaa, 2018). Thus, research conducted by Danış et al., 2023 suggested that geranium oil treatment has a beneficial effect in reducing this organ damage.

## 5 Conclusion

The results of this investigation highlight the important protective effects of geranium oil against the hepatotoxicity and renal toxicity that cefotaxime induces in rats. The detrimental effects of cefotaxime were significantly reduced by geranium oil, as demonstrated by the significant declines in liver enzyme activities in serum, renal markers, and other biochemical damage indicators. Additionally, geranium oil increased the levels of tissue biomarkers that are protective, such as glutathione, NrF2, albumin, catalase, Beclin 1, and catalase, indicating that it plays a part in preventing oxidative stress and cellular damage. The potential of geranium oil as a therapeutic agent was highlighted by histopathological and immunohistochemical experiments that confirmed its protective effects on liver and renal tissues. These findings suggest that geranium oil may be a useful adjuvant in reducing the side effects of cefotaxime, providing a viable method of treating drug-induced toxicity.

## 6 Recommendation

More studies are needed for exploring the geranium oil active ingredients effective role and pathways responsible for hepato renal protective influence. Furthermore, examining dose-response interactions at varying concentrations of both geranium oil and cefotaxime would enhance understanding of its efficacy and safety profile. Future research should also consider extending the observation period, including both sexes, increasing sample sizes, and evaluating the translational potential of these findings in human therapeutic trials. Additionally, comprehensive safety assessments of long-term geranium oil administration, as well as studies exploring its protective effects against other antibiotics known to induce hepatotoxicity and nephrotoxicity, would provide valuable insights for its potential therapeutic applications.

## Data Availability

The datasets presented in this study can be found in online repositories. The names of the repository/repositories and accession number(s) can be found in the article/supplementary material.
